# Establishment of the microscope incubation system and its application in evaluating tumor treatment effects through real-time live cellular imaging

**DOI:** 10.3389/fbioe.2024.1447265

**Published:** 2024-08-16

**Authors:** Haiyang Yan, Tong Wu, Xinlu Li, Zhengyang Feng, Mingfeng Ge, Lixing Zhang, Wen-Fei Dong

**Affiliations:** ^1^ School of Biomedical Engineering (Suzhou), Division of Life Sciences and Medicine, University of Science and Technology of China, Hefei, China; ^2^ CAS Key Laboratory of Biomedical Diagnostics, Suzhou Institute of Biomedical Engineering and Technology, Chinese Academy of Science (CAS), Suzhou, China; ^3^ Department of Oncology, The Second Affiliated Hospital of Soochow University, Suzhou, China; ^4^ Zhengzhou Institute of Biomedical Engineering and Technology, Zhengzhou, China

**Keywords:** microscope incubation system, long-term imaging, PID algorithm, organoid culture, live cell microscope

## Abstract

**Introduction:** Long-term imaging of live cells is commonly used for the study of dynamic cell behaviors. It is crucial to keep the cell viability during the investigation of physiological and biological processes by live cell imaging. Conventional incubators that providing stable temperature, carbon dioxide (CO_2_) concentration, and humidity are often incompatible with most imaging tools. Available commercial or custom-made stage-top incubators are bulky or unable to provide constant environmental conditions during long time culture.

**Methods:** In this study, we reported the development of the microscope incubation system (MIS) that can be easily adapted to any inverted microscope stage. Incremental PID control algorithm was introduced to keep stable temperature and gas concentration of the system. Moreover, efficient translucent materials were applied for the top and bottom of the incubator which make it possible for images taken during culture.

**Results:** The MIS could support cell viability comparable to standard incubators. When used in real time imaging, the MIS was able to trace single cell migration in scratch assay, T cell mediated tumor cells killing in co-culture assay, inflation-collapse and fusion of organoids in 3D culture. And the viability and drug responses of cells cultured in the MIS were able to be calculated by a label-free methods based on long term imaging.

**Discussion:** We offer new insights into monitoring cell behaviors during long term culture by using the stage adapted MIS. This study illustrates that the newly developed MIS is a viable solution for long-term imaging during in vitro cell culture and demonstrates its potential in cell biology, cancer biology and drug discovery research where long-term real-time recording is required.

## 1 Introduction

*In vitro* cell cultures have been widely used in various fields in academia and industry, such as cell biology and drug discovery research; they have been proven to be valuable tools for investigating molecular mechanisms and cell physiology. In cancer research, cell cultures have been developed to elucidate the fundamental mechanisms of cancer initiation, progression, and drug responses (Vallette et al., 2019). Cell-based assays are used to screen large numbers of compounds quickly and efficiently, providing valuable information on the potencies and specificities of potential drugs (Gregory et al., 2016). Cell cultures have also been used to study virus–host interactions and develop antiviral vaccines (Talebipour et al., 2022). Moreover, *in vitro* three-dimensional cell models that physiologically mimic intact organs are more accurate and predictive for studies on cell–cell interactions, drug testing, and cancer characteristics (Jubelin et al., 2022). Overall, *in vitro* cell cultures are becoming increasingly important with developments in gene editing, reprogramming, and *in vitro* differentiation.

In recent years, the field of cell culturing has seen rapid developments toward study of dynamic cellular behaviors. Monitoring cell growths over time and capturing their behaviors in real time enable researchers to better understand cellular processes, such as cell proliferation, differentiation, and migration (Svensson et al., 2018; Scanlon et al., 2022). This powerful tool helps deepen the understanding of cellular behaviors and their underlying mechanisms, revolutionizing the field of cell biology (Cuny et al., 2022). However, maintaining vital conditions such as stable temperature of approximately 37°C, 5% concentration of CO_2_ to stabilize the pH of the cell culture medium, and relative humidity of about 90%–95% to minimize water evaporation during long-term imaging can be challenging as most conventional inverted microscopes are not adaptable to such imaging conditions (Killian et al., 2016).

Numerous technological innovations have enhanced the utility of live imaging, including the development of microscopes for use in incubators and incubators for use in microscopy systems. However, cell incubators are often incompatible with many screening tools and may destroy some of the optical imaging elements, limiting the experimental possibilities. Another impediment is optimizing the size of the microscope for use in an incubator, especially the fluorescence microscope. There have been reported trends toward the development of smaller incubators that can be positioned atop the microscope stage for long-term observations (Talebipour et al., 2024). Maintaining a constant cellular environment is important; further, the cells should be grown in culture media in a carbon dioxide incubator. Temperature control is a critical criterion for most cultured cells, where a change of even 0.5°C can significantly affect embryo development and division (Walters et al., 2020). Changes in the pH can cause changes in the cellular morphologies (Lo et al., 1994), and this has been demonstrated for *in vitro* porcine embryo cultures (Ozawa et al., 2006). Suboptimal gas concentrations can alter the expansion, differentiation, and transcription profiles of the cells (Moussavi-Harami et al., 2004; Martin-Rendon et al., 2007). Hence, mini incubators have been specifically designed to provide a stable and controlled environment for cell growth while being compact and adaptable to different microscopes.

Temperature control is often achieved using peripheral sources of heated air, infrared radiation, metal heating plates controlled by thermistors, or transparent thin coatings of electrically conductive metal oxides on coverslip surfaces. The incubation systems used at present often experience significant temperature fluctuations and uneven temperature distributions. These occur because the microscope stage, frame, and objectives act as heat sinks and offset the heating system used (Jensen, 2013). Moreover, the environment within the incubator may be incompatible with the electronic components. These challenges reinforce the need for incubation systems that provide stable and controlled environments that enable continuous, non-invasive monitoring of cell cultures without compromising the stability and integrity of the culture environment.

Closed-loop and open-loop control algorithms are two commonly used approaches to achieve temperature and gas control; closed-loop control algorithms are known to be stronger and more robust than open-loop methods. The proportional integral derivative (PID) control algorithm is one of the widely used closed-loop control approaches to achieve precise temperature control (Jamil et al., 2022). Both incremental and fuzzy PID methods have been demonstrated to allow precise temperature control (Wang et al., 2020; Li et al., 2023), which is why it has been used for temperature control in the development of the mini incubator.

In this study, we developed a microscope incubation system (MIS) based on the high-precision incremental PID algorithm to maintain a stable temperature of 37°C ± 0.19°C and CO_2_ gas concentration of 5% ± 0.13% inside the mini incubator. The external gas was premixed by forced convection to improve the anti-interference ability and robustness of the gas distribution module. In addition, sterile water was added to maintain the high relative humidity (RH) of the environment (>90%) via natural evaporation. A splicing program was applied with different thermally conductive materials to maintain the heat transfer efficiency and uniformity in the incubator, which also helped to reduce the heat loss from the system. All these elements were compacted into a box-like structure of dimensions 160 mm × 130 mm × 29 mm, which is suitable for most of the available converted microscope stages and cell culture dishes or plates. Based on the above design, the proposed mini incubator system provides a stable and reliable environment for *in vitro* cell culture on a microscope stage. To evaluate the accuracy and functionality of the proposed MIS, we performed cell and organoid cultures; we noted that the resulting cell viability and proliferation in the MIS were comparable to those achieved with a conventional incubator. Moreover, the MIS could be used to monitor cell migration, drug responsibility, and cell killing abilities of the immune cells. Overall, the newly developed MIS showed potential for cell culturing, enabling non-invasive and real-time monitoring of cellular behaviors with ease and precision.

## 2 Materials and methods

### 2.1 MIS design and setup

Herein, we develop an MIS consisting of an incubator, a main control module, a temperature control module, and a gas control module. The incubator (Suzhou Xinsheng Shell Model Technology Co., Ltd.) had a body made of 6061 aluminum alloy, with a polyphenylene sulfide (PPS) body baseplate, a PPS incubator body cover, a replaceable aluminum alloy baseplate for the cell culture plates or dishes, quartz glass windows on the body cover, and an aluminum alloy baseplate. The entire incubator had dimensions of 160 mm × 130 mm × 29 mm, which made it suitable for placement on a microscope stage (HDS-IH-75110SN, Heidstar Co., Ltd.). The high thermal conductivity of the aluminum alloy enables rapid and uniform temperature transfer within the incubator, while the relatively low thermal conductivity of PPS reduces the heat loss from the incubator.

The main control module comprised a host computer and a STM32 microcontroller. The host computer was used to display, store, and set the parameters of the system, while the microcontroller was to used store and run the control program. The host computer was automated using LabView 2018 software (National Instruments, United States), and the controller program was based on the PID algorithm. The temperature control module comprised a heating drive circuit, a temperature measurement circuit, and ADS8688 analog-to-digital converter (ADC, Texas Instruments, United States), PT1000 temperature sensors (Wuhan Xunce Sensor Technology Co., Ltd.), and heating elements (Supplementary Figures S1, S2).

The working of the temperature control module is as follows: the resistance of the temperature sensor PT1000 was tested using a temperature measurement circuit and converted to an analog signal in the form of a voltage; this analog signal was next transmitted to the ADC and converted to a digital temperature signal. Then, the temperature signal was sent to the microcontroller through the SPI interface. After receiving the temperature signal, the power control of the heating element was achieved using the microcontroller through the PID algorithm and output to the heating drive circuit in the form of a pulse width modulated (PWM) wave with a certain duty cycle. This signal was then applied to the corresponding position heating elements (i.e., heating elements on both sides of the incubator are polyimide heating films, Dongtai Kedeman Electric Heating Technology Factory). The heating elements under the top glass are transparent films made of indium tin oxide (ITO, Shenzhen Kuchi New Energy Technology Co., Ltd.) that are used to adjust the input power in real time.

The gas control module comprised a 4-L CO_2_ cylinder (Suzhou Jinhong Gas Co., Ltd.), a gas mixing tank (Zhengzhou Yanke Instrument Equipment Co., Ltd.), an E-series mass flow controller (MFC, Qingdao Xinsheng Micro Nano Electronic Technology Co., Ltd.), a SprintIR-CO_2_ sensor (Gas Sensing Solutions, United Kingdom), and an air pump (Nidec, Japan). The gas control process is as follows: air was absorbed into the air pump and CO_2_ was provided from a gas cylinder. Then, the air and CO_2_ passed through the MFC and were compressed into the closed gas mixing tank for premixing. The internal gas concentration in the gas mixing tank was detected using a sensor in a timely manner and transmitted to the microcontroller. The gas flow was then adjusted using the MFC based on the PID algorithm. Finally, a set concentration of gas was continuously injected into the incubator through the air inlet.

### 2.2 Temperature and gas control

In this system, a high-precision incremental PID control algorithm was used to maintain the ambient temperature and gas concentration at stable levels to realize low fluctuations of the ambient parameters of the incubator. The incremental PID control algorithm was improved using the positional PID algorithm, which eliminated the cumulative error in the positional PID algorithm and further improved the control accuracy. The formula of the incremental PID algorithm is as follows: $\Delta u\left(k\right)=u\left(k\right)-u\left(k-1\right)=K_{p}\left[e\left(k\right)-e\left(k-1\right)\right]+K_{i}e\left(k\right)+K_{d}\left[e\left(k\right)-2e\left(k-1\right)+e\left(k-2\right)\right]$ , where $K_{p}$ , $K_{i}$ , and $K_{d}$ represent the coefficients of proportionality, integration, and differentiation, respectively, $e\left(k\right)$ is the difference between the set and current values of the controlled parameter, $u\left(k\right)$ is the actual control parameter of the actuator, $u\left(k-1\right)$ is the previously calculated value of the actual control parameter of the actuator, $e\left(k-1\right)$ is the difference between the controlled parameter and set value calculated in the previous step, and $e\left(k-2\right)$ is the difference between the controlled parameter and set value calculated two steps before.

### 2.3 Gas mixing simulation

The gas sensor could not be placed within the incubator owing to its limited size, and the premixing gas distribution program was set outside the incubator. A fan was introduced into the mixing tank of the gas distribution module to force convection, which helped achieve rapid gas mixing and stabilization. To investigate the CO_2_ homogeneity of the gas flow field in the gas mixing tank, a CFD simulation of the gas flow field was conducted in ANSYS Fluent (ANSYS, United States).

The CO_2_ and air were separately passed into the gas mixing tank. Since these two gases are compatible, the gas mixture model could be simulated using multicomponent flow calculations, where different continuity equations were used for each gas while the momentum and energy equations were shared. The differential forms of the continuity, momentum, and energy equations are as follows.

The continuity equation is given by $\frac{\partial \rho }{\partial t}+\nabla ∙\left(\rho \vec{V}\right)=0$ , where $\frac{\partial \rho }{\partial t}$ denotes the increase in mass at a point and $\nabla ∙\left(\rho \vec{V}\right)$ represents the mass flowing out of that point.

The momentum equation is given by $\frac{D\vec{V}}{DT}=\vec{f_{b}}-\frac{1}{\rho }\nabla p+\frac{\mu }{\rho }\nabla^{2}\vec{V}+\frac{1}{3}\frac{\mu }{\rho }\nabla \left(\nabla ∙\vec{V}\right)$ , where $\frac{D\vec{V}}{DT}$ is the inertial force term, $\vec{f_{b}}$ is the volumetric force term, $-\frac{1}{\rho }\nabla p$ is the differential pressure force term, and $\frac{\mu }{\rho }\nabla^{2}\vec{V}+\frac{1}{3}\frac{\mu }{\rho }\nabla \left(\nabla ∙\vec{V}\right)$ is the viscous force term.

The energy equation is given by $\rho \frac{D}{Dt}\left(\hat{u}+\frac{V^{2}}{2}\right)=\rho \vec{f_{b}}∙\vec{V}+\nabla ∙\left(\vec{V}∙\tau_{ij}\right)+\nabla \left(\lambda \nabla T\right)+\rho \dot{q}$ , where $\rho \frac{D}{Dt}\left(\hat{u}+\frac{V^{2}}{2}\right)$ is the total energy change of the fluid microcluster, $\rho \vec{f_{b}}∙\vec{V}$ is the work done on the fluid microcluster by the volume force, $\nabla ∙\left(\vec{V}∙\tau_{ij}\right)$ is the work done on the fluid microcluster by the surface force, $\nabla \left(\lambda \nabla T\right)$ is the heat received by the fluid microcluster from the external environment through heat conduction, and $\rho \dot{q}$ is the heat received by the fluid microcluster from the external environment through radiation.

### 2.4 Measurements of temperature, gas, and humidity in the MIS

Once the system was set up, the performances related to temperature control accuracy and uniformity, gas control accuracy, and humidity accuracy were tested at room temperature. Among these, the data fluctuations in the bottom center area were tested for the temperature control performance and humidity, and the gas concentration fluctuations in the gas mixing tank were tested for the gas control performance. The sensors used for the temperature, gas, and humidity measurements were the PT1000, SprintIR-CO_2_, and SHT35 (Sensirion, CH) devices, respectively.

### 2.5 Cell cultures

The MC38 cell line was received as a gift from Dr. Minxuan Sun (SIBET, CAS). The cancer-associated fibroblasts (CAFs) and HeLa cell line were obtained from lab freezing. All cell lines were cultured in Dulbecco’s modified Eagle’s medium (DMEM, Servicebio) with 10% fetal bovine serum (FBS, Servicebio) and 1% penicillin/streptomycin (Gibco) and incubated at 37°C in a 5% CO_2_ humidified atmosphere incubator. For all the experiments, the cell lines were grown to 70%–80% confluence.

### 2.6 Human colorectal cancer (CRC) organoids cultured in MIS

Primary CRC tissues were obtained from patients who underwent surgical resections at the Second Affiliated Hospital of Soochow University. These studies were conducted in accordance with the ethical guidelines and the Declaration of Helsinki. Informed consent was obtained from all the participants. The organoids were cultured according to the procedures of Wang et al. (2022). Briefly, fresh tumor tissues were cut into small pieces, washed with ice-cold phosphate buffered saline (PBS) containing 1% penicillin/streptomycin, 0.25 μg/mL of amphotericin B (Solarbio), and 10 μg/mL of gentamicin (Solarbio) at least three times. The tissues were subsequently digested using DMEM containing 10% FBS, 1.5 mg/mL of collagenase II (Solarbio), 500 U/mL of collagenase II (Solarbio), 20 μg/mL of hyaluronidase (Sigma), and 10 μM of Y27632 (Biogems) for 30–60 min at 37°C. After digestion, the dissociated tissues were passed through a 70-μm cell strainer and centrifuged at 150*g* for 5 min. The cell pellets were suspended in BME (Reduced Growth Factor, R&D Systems) and dispensed into a 3.5 mm cell dish. After incubation at 37°C for 20 min, the CRC organoid culture medium was added, which contained advanced DMEM/F12 (Gibco), 1× B27 (Gibco), 1% penicillin/streptomycin, 100 μg/mL of normocin (InvivoGen), 10 mM of HEPES (Gibco), 1× GlutaMAX (Gibco), 10 mM of nicotinamide (Sigma), 100 nM of prostaglandin E2 (Sigma), 1.25 mM of N-acetyl cysteine (Sigma), 0.5 μg/mL of R-spondin (Sino Biological), 10 nM of gastrin I (Sigma), 50 ng/mL of EGF (Stem Cell), 100 ng/mL of noggin (Sino Biological), 10 μM of Y27632, 3 μM of SB202190 (Sigma), and 0.5 μM of A83-01 (MedChemExpress). Then, the organoids were cultured in the MIS or conventional incubator and images were acquired daily or every 15 min.

### 2.7 Cell viability assay

Cell viability was measured by the water-soluble tetrazolium salt-1 (WST-1, Beyotime) assay and lactate dehydrogenase (LDH, Yeasen) assay. The cells were then cultured in the conventional incubator or MIS for a defined amount of time. For the WST-1 assay, the cell culture medium was replaced with fresh medium containing 10% WST-1. After incubation at 37°C for 30 min, the OD450 value of the medium was analyzed. For the LDH assay, the OD490 value of the treated cell culture medium was analyzed according to manufacturer instructions.

### 2.8 Calcein-AM/PI staining

Calcein-AM/PI staining was performed to test the ratio of live to dead cells. Briefly, the cells or organoids were stained using reagents with the Calcein-AM/PI Double Staining Kit (Yeasen) according to manufacturer instructions after culturing in the conventional incubator or MIS. Fluorescent images were then captured using an inverted fluorescence microscope (Nikon).

### 2.9 *In vivo* tumorigenicity assay

Animal experiments were performed according to the National Institutes of Health Guide for the Care and Use of Laboratory Animals and were approved by the Biological Research Ethics Committee of the Chinese Academy of Sciences. Here, 7–8-week-old male C57BL/6J mice (SPF Biotechnology, China) were maintained in a pathogen-free environment under 12/12 h light/dark cycle. Following 1 week of acclimatization, the mice were subcutaneously injected with 1 × 10^6^ MC38 cells cultured in the conventional incubator or MIS (four mice per group). The lengths (L) and widths (W) of the tumors were measured twice a week, and the tumor size was calculated as L × W^2^/2 (Chao et al., 2021).

### 2.10 T-cell-mediated tumor cell toxicity assay

The C57BL/6J mice were subcutaneously injected with MC38 cells, and cytotoxic T cells were obtained from the spleens of the tumor bearing mice on day 21 after injection. The spleens were ground with a syringe bolus and passed through a 70-μm cell strainer. After erythrocyte lysis, the suspension was centrifuged at 200*g* for 10 min. To enrich the T cells, RPMI-1640 medium supplied with 10% FBS, 20 mM of HEPES, 1× GlutaMAX, 1% penicillin/streptomycin, 50 μM of 2-mercaptoethanol (Sigma), and 10 ng/mL of IL-2 (Novoprotein) were used for the cell culture.

For coculturing the T cells and tumor cells, 3 × 10^5^ MC38 cells labeled with 20 μM of 3,3′-dioctadecyloxacarbocyanine perchlorate (DIO, Beyotime) were plated in a 3.5 cm cell culture dish. After cell adhesion, 6 × 10^6^ T cells labeled with 10 μM of 1,1′-dioctadecyl-3,3,3′,3′-tetramethylindocarbocyanine perchlorate (DIL, Beyotime) were added. Then, the cell culture dish was transferred to the MIS and images were acquired using the microscope every 15 min. The cytotoxicity of the T cells on the tumor cells was measured using the LDH releasing assay using the following formula: cytotoxicity (%) = [(OD experiment group – OD target spontaneous group)/(OD maximum group – OD target spontaneous group)]×100% (Li et al., 2022).

### 2.11 Label-free cell viability and antidrug activity analyses

To evaluate the cell proliferation and cell viability, 3 × 10^5^ cells were plated in a 3.5 cm cell culture dish and incubated in the MIS. Images were then acquired, and the cell confluence was quantified using ImageJ software (NIH, United States). Cell viability was calculated by the following formula: cell viability (%) = (confluence_drug group_/confluence_control group_) ×100%. As control, the cell viability was tested via the WST-1 assay and calculated as follows: cell viability (%) = (OD_drug group_/OD_control group_) ×100%.

To test the antidrug activities of the tumor cells, dimethyl sulfoxide (DMSO) or different concentrations of chemotherapeutic drugs were added to the culture medium. Images were then captured, and the cell confluence was quantified using ImageJ software (NIH, United States). To evaluate the effects of different drugs on cell growth, the cell growth rate (GR) was calculated according to the following equation (Hafner et al., 2016): GR = $2^{\frac{\log2\left(x_{\left(c\right)}/x_{0}\right)}{\log2\left(x_{ctrl}/x_{0}\right)}}-1$ , where x_(c)_ is the confluence of the cells treated with drugs, x_ctrl_ is the confluence of the untreated control cells, and x_0_ is the cell confluence before treatment.

### 2.12 Scratch assay recorded by the MIS

To validate whether the MIS could be used to monitor cell migration, a scratch assay was performed with the MIS. In brief, the cells were labeled with 10 μM of DIL and cultured in the MIS. Once the cell confluence reached 80%, scratches were made in the cell culture dish using 200 μL pipette tips; then, fresh medium without FBS was added to the cell culture dish, followed by culturing in the MIS. The scratches were recorded by the microscope in real time. The uncovered area and locations of the cells were analyzed using ImageJ software, and the migration rate (%) was calculated as follows: migration rate (%) = [(initial width − width after incubation)/initial width] ×100%. The trajectories of the cell movements were then plotted using MATLAB (The MathWorks, Natick, MA, United States).

### 2.13 Statistical analysis

The numerical data were presented as means ± standard errors of the means (SEMs) and were subjected to the unpaired t-test using GraphPad Prism 8.0 (GraphPad Software, United States). Statistical significance between the treated and control groups was examined using the Student’s t-test. A value of *p* < 0.05 or *p* < 0.01 was considered to be statistically significant, and *p* > 0.05 (ns) indicated that the difference was not significant.

## 3 Results

### 3.1 Development of the MIS

**FIGURE 1:**
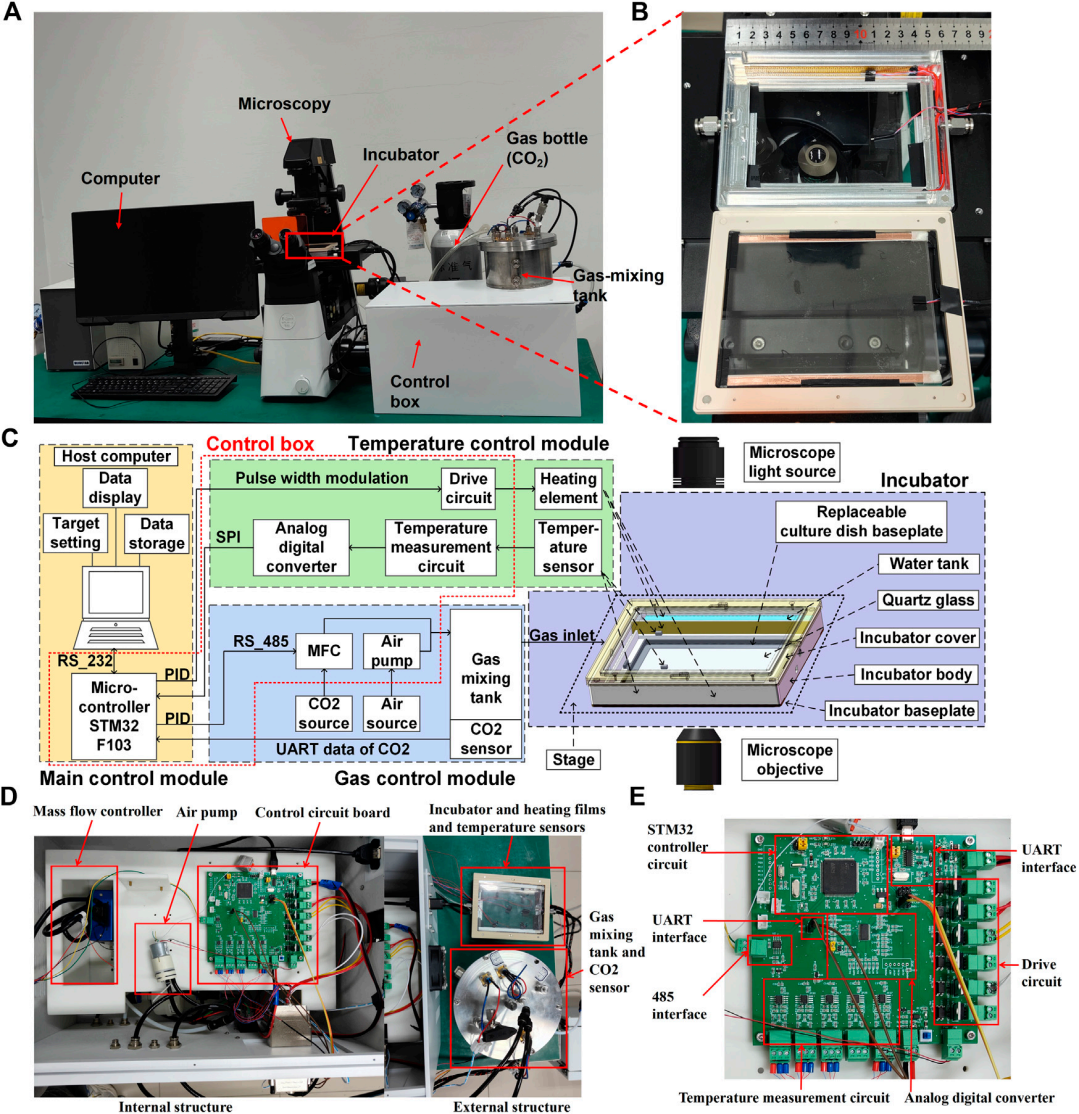
Systematic composition of the microscope incubation system (MIS) showing the **(A)** exterior components, **(B)** picture of the incubator, **(C)** schematic of the control relationships among the internal components, **(D)** internal and external structures of the control box, and **(E)** detailed structure of the control circuit board.

We designed and manufactured a thermostatic incubator that could be placed on an inverted microscope stage. This device, along with the microscope, enabled real-time imaging of live cells. The exterior components of the MIS are shown in Figure 1A and include carbon dioxide cylinders, gas mixing tanks, a control box, a host computer, an incubator, and a microscope for imaging. The enlarged view of the incubator is shown in Figure 1B. As shown in Figure 1C, the components of the MIS are the main control module, temperature control module, gas control module, and incubator. To ensure that the system is more integrated, the microcontroller in the main control module, temperature module (except the heating element and temperature sensor), and gas control module (except the air source, CO_2_ source, and gas mixing tank) were compacted in a control box, as shown in Figure 1D. The heating element and temperature sensor were compacted into the incubator module. The detailed control circuit board is shown in Figure 1E.

**FIGURE 2:**
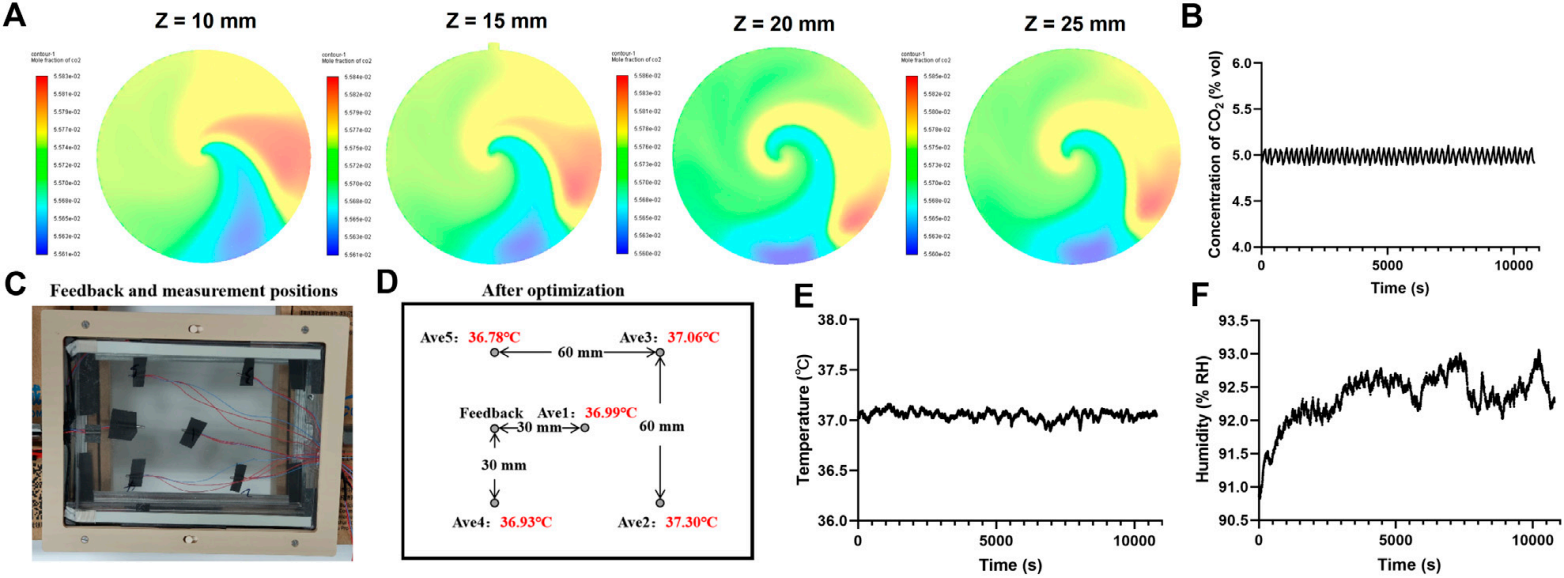
Characterization of the microscope incubation system (MIS). **(A)** Simulation results of CO_2_ concentration in the gas mixing chamber along four planes (Z = 10 mm, Z = 15 mm, Z = 20 mm, and Z = 25 mm) showing the corresponding CO_2_ mass fraction cloud plots. **(B)** Actual measured value of the internal CO_2_ concentration. **(C)** Feedback and measurement positions for the temperature. **(D)** Average temperature at each measurement position within 30 min. Actual measured values of the internal **(E)** temperature and **(F)** humidity over 3 h of recording after stabilization of the system.

In detail, the cover and base of the incubator were made of PPS with quartz glass for better sealing and clear imaging of the cells. Water tanks were arranged on both sides of the incubator and filled with water during cell culturing. Natural water evaporation was maintained to ensure proper humidity for cell growth inside the incubator. Two polyimide heating elements were placed close to the side walls of the water tank to maintain a stable temperature and enhance water evaporation. The main heater was a transparent ITO film placed under the top quartz glass, allowing clear observation of the cells. PT1000 temperature sensors were attached to monitor and control the side heating films in real time. The temperature sensor used to monitor and control the top heating film in real time was placed at the bottom of the incubator; specifically, it was placed at the center of the side of a 60 × 60 mm rectangle centered on the bottom surface, as shown in Figure 2C. Multiple heating films were used together to maintain stable temperature in the area where the culture dish was placed.

### 3.2 Characterization of the MIS

To investigate whether the incubator could be used for cell culturing, its key characteristics were evaluated. The numerical calculation fluid domain model was established in ANSYS Space Claim, as shown in Supplementary Figure S3. The fluid domain size was 150 mm × 75 mm. The total flow rate of the target gas CO_2_ and diluted gas air was set to 200 sccm. Based on the pipe diameter, the fluid velocity through the air mixing chamber was set to 0.1 m/s. Since the required target gas concentration was 5% by volume, the required air flow rate was 0.095 m/s and CO_2_ flow rate was 0.005 m/s according to the following calculation formula of the gas mixing ratio: $C_{c}=\frac{L_{c}∙C_{0}}{L_{A}+L_{c}}$ , where $C_{c}$ is the target concentration of CO_2_, $L_{c}$ is the flow rate of CO_2_, $C_{0}$ is the gas source concentration of CO_2_, and $L_{A}$ is the flow rate of air.

To ensure uniformity of gas mixing in the simulation experiment, five planes (X = 0 mm, Z = 10 mm, Z = 15 mm, Z = 20 mm, Z = 25 mm) were captured to display the CO_2_ mass fraction cloud image (Supplementary Figure S3B, Figure 2A). The outlet was at Z = 15 mm, and the sensor monitored the gas at Z = 20 mm. The CO_2_ mass fraction at each plane closely matched the input gas ratio, confirming the suitability of this model for controlling the gas ratio. The flow rate of the air pump in the system was 180 sccm, and the flow rate range of the MFC was 0–20 sccm, with the total flow rate calculated as 180–200 sccm. The gas concentration was measured for 3 h, and the degree of fluctuation of the gas concentration in the system was 5% ± 0.13% by volume (Figure 2B).

The system parameters were tested in accordance with the methods described in Section 2 (YY/T 1621–2018 medical carbon dioxide incubator and GB/T 28851–2012 biochemical incubator technical conditions were used as control). The MIS was operated at room temperature. The target temperature used to control the top heating film was set at 37°C, and the temperature of the side heating film was controlled at 37.5°C. The temperature was measured at the center and four vertex positions of a 60 × 60 mm^2^ rectangle at the bottom for more than 30 min (Figure 2C). The temperature uniformity of this area was calculated as 37°C ± 0.26°C (Figure 2D), and the temperature accuracy was calculated as 37°C ± 0.19°C (Figure 2E). The humidity of the system was also measured for 3 h, and the data showed that the humidity was stable at >90% RH (Figure 2F). The measured characteristics of the MIS suggest its potential use as a microscope incubator for real-time imaging.

### 3.3 Cell viability and function maintenance in the MIS

**FIGURE 3:**
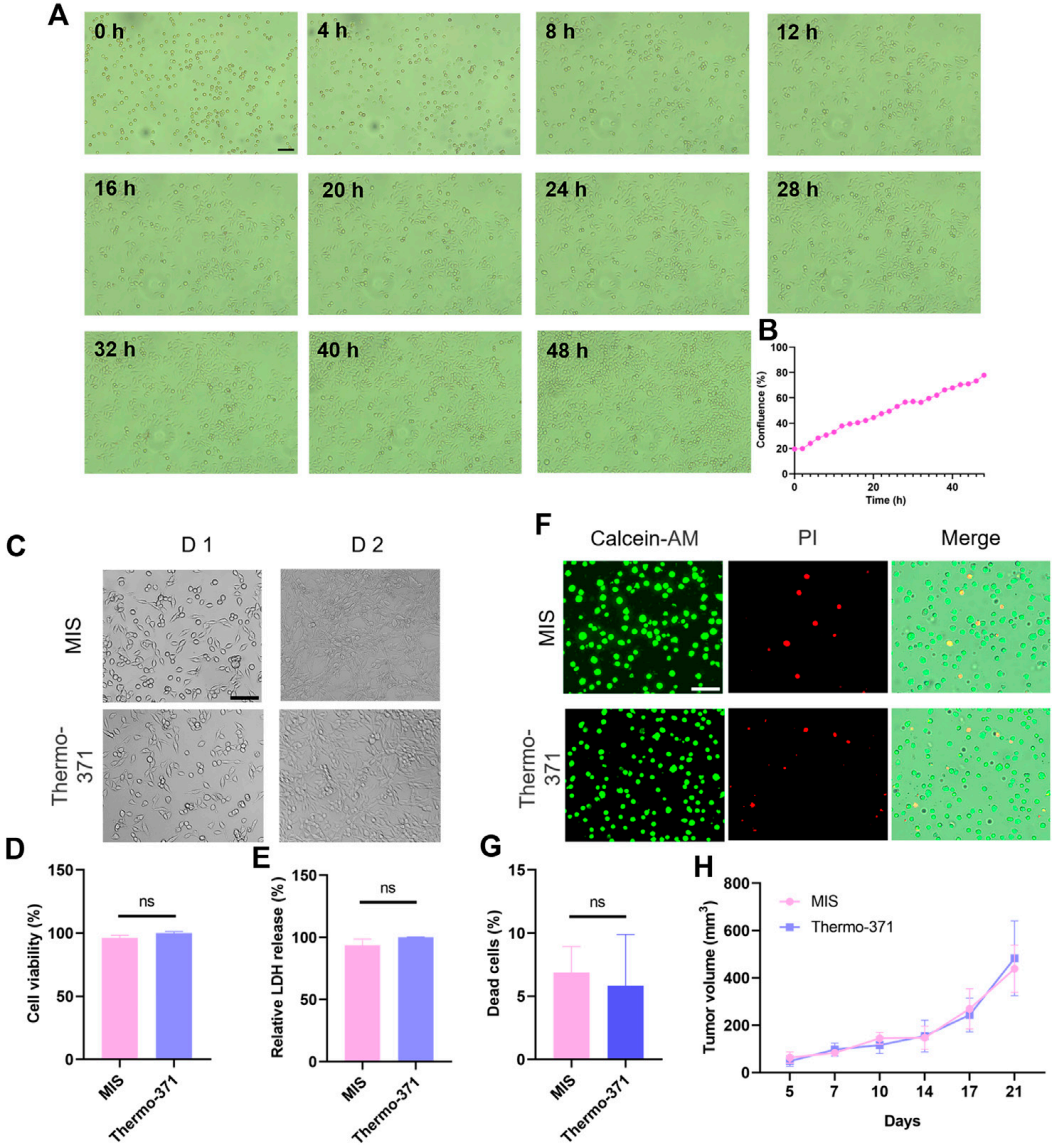
Comparisons of cell growth and cell viability upon culturing in the microscope incubation system (MIS) and a conventional incubator. **(A)** Bright-field images of HeLa cells cultured in the MIS captured at different time points. **(B)** Label-free calculation of cell growth based on cell confluence. **(C)** Representative bright-field images of the MC38 cells cultured in the MIS or Thermo-371 incubator on different days. Cell viability comparisons between cells cultured in the MIS or Thermo-371 incubator by **(D)** WST-1 and **(E)** LDH releasing assays. **(F)** Representative fluorescent images of the live (green) and dead (red) cells after culturing in the MIS or Thermo-371 incubator. **(G)** No significant differences are seen in the proportions of dead cells (*p* > 0.05) between cells cultured in the MIS and Thermo-371 incubator. **(H)** Tumor growth rates in the C57BL/6J mice injected with MC38 cells cultured in the MIS and Thermo-371 incubator (n = 4 mice per group). Scale bar: 100 μm, ns: *p* > 0.05. WST-1: water-soluble tetrazolium salt-1; LDH: lactate dehydrogenase.

Next, we investigated whether the MIS could support cell cultures, which is the precondition in this study for real-time imaging. The MIS was placed on an inverted microscope stage to capture the real-time cell growths. To measure the cell growth with the MIS, HeLa cells were plated in a 3.5 mm dish and images were acquired every 2 h, which showed the gradual growths of the cells (Figures 3A,B). Then, MC38 cells were cultured in both the MIS and the conventional Thermo-371 incubator. After 2 days, similar cell morphologies were observed in both systems, demonstrating that the MIS maintained appropriate cell growth (Figure 3C). The cell viability was then evaluated by the WST-1 assay, and no significant differences were observed between the cells cultured in the MIS and Thermo-371 incubator (Figure 3D). The LDH releasing assay also showed similar cell viabilities when cultured in the MIS and Thermo-371 incubator (Figure 3E). Furthermore, calcein-AM/PI staining was performed to evaluate the cell death. As shown in Figures 3F,G, a majority of live cells were observed when cultured in the MIS or Thermo-371 incubator, and the percentage of dead cells showed no significant difference (*p* > 0.05) between the two systems. HeLa cells cultured in the MIS were also analyzed, and calcein-AM/PI staining showed a few dead cells (Supplementary Figure S4A). Interestingly, there was less LDH released from the cells cultured in the MIS than from those cultured in the Thermo-371 incubator (Supplementary Figure S4B). Frequent removal of the cells from the traditional incubator for observation could affect cell viability, whereas the MIS allowed uninterrupted long-term cultivation. Then, the MIS was used to culture the primary cells. CAFs isolated from pancreatic cancer cells were cultured in the MIS, and the cell viability was maintained well over the 48-h culture period (Supplementary Figure S4C). Furthermore, the tumor generating abilities of the MC38 cells cultured in the MIS were tested through a transplantation assay; no significant differences (*p* > 0.05) were found in the tumor growths between cells grown in the MIS and those grown in the Thermo-371 incubator (Figure 3H). To summarize, the cell viabilities of the cell lines and primary cells are well maintained during culturing in the proposed MIS, which is comparable to the results achieved with the commercially available Thermo-371 incubator.

### 3.4 MIS can be used to culture and record the growth of organoids

**FIGURE 4:**
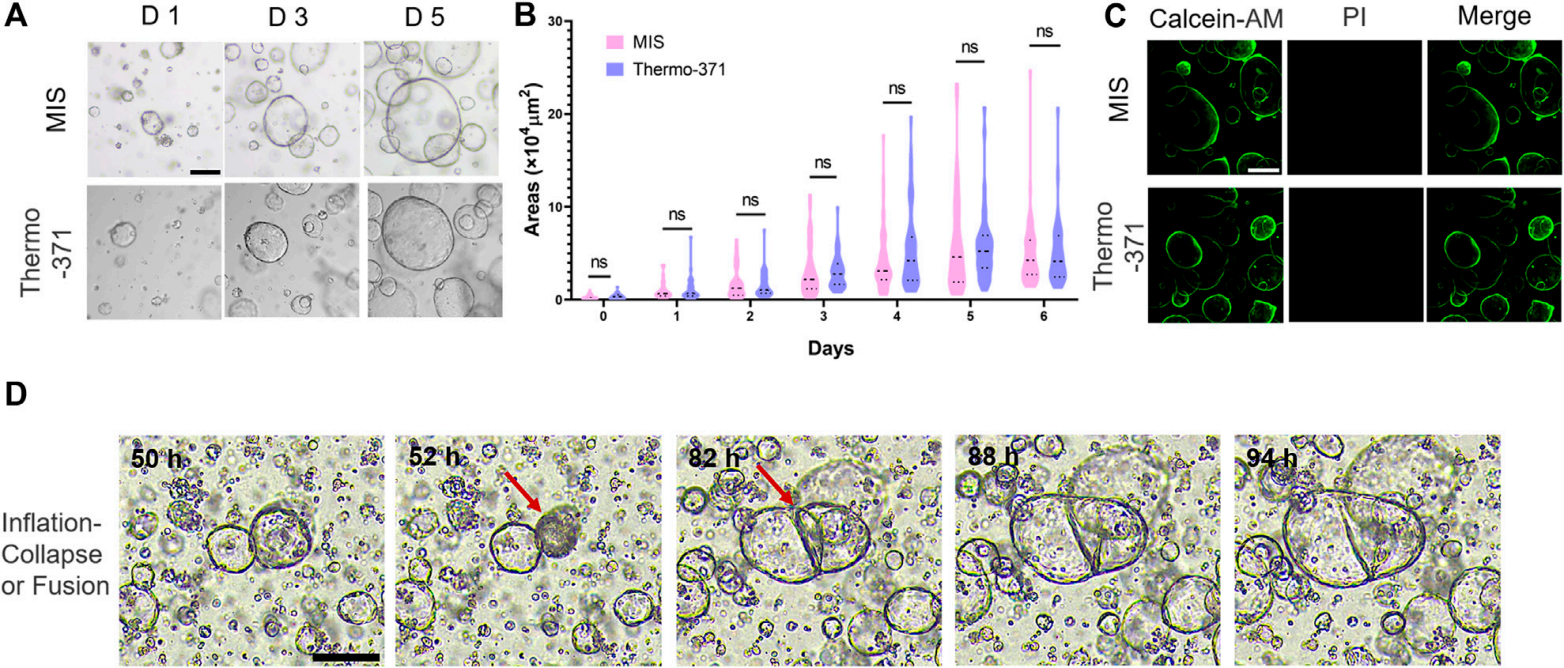
The microscope incubation system (MIS) maintains the tumor organoid growths and can record rapid changes in the organoid morphologies and structures. **(A)** Representative bright-field images of colorectal cancer (CRC) organoids cultured in the MIS and Thermo-371 incubator on days 1, 3, and 5. **(B)** The sizes of the CRC organoids cultured in the MIS and Thermo-371 incubator show no significant differences (*p* > 0.05) on different days. **(C)** Representative fluorescent images of the live (green) and dead cells (red) in organoids cultured in the MIS and Thermo-371 incubator. **(D)** Images of representative organoids undergoing inflation–collapse and fusion processes. Scale bar: 200 μm. ns: *p* > 0.05. Calcein-AM: calcein acetoxymethyl ester; PI: propidium iodide.

Organoids are 3D tissue cultures grown from stem cells and have been used to study the cellular heterogeneities, structures, and functions of tissues *in vitro* (Corrò et al., 2020; Huang et al., 2023). Herein, we investigated whether the proposed MIS could be used for such 3D cultures. CRC organoids cultured from patient tissues were grown in the MIS for at least 7 days, and images were automatically acquired every 15 min by the microscope in the MIS setup. The organoids cultured in the MIS were bright and plump, similar to those cultured in the Thermo-371 incubator (Figure 4A; Supplementary Figure S6), and there were no significant differences (*p* > 0.05) in the organoid sizes between those cultured in the MIS or Thermo-371 incubator (Figure 4B). On day 7, the organoids were labeled with calcein-AM/PI, and few dead cells were found in organoids cultured in both systems (Figure 4C). Thus, cell growth was adequate in the MIS for both two-dimensional (2D) and 3D cultures.

As the images were obtained every 15 min for more than 7 days, videos were created to record the real-time growths of the organoids (Supplementary Video S1). Most organoids grew larger gradually, while some of the organoids, especially the hollow ones, collapsed and shrank during the culturing (Figure 4D). We noted that some of the organoids merged when they were close and attached to each other, resulting in larger structures; this process could be overlooked in traditional incubators without real-time imaging as the process of collapsing or merging usually happens over a short time. This may also explain the sizes of some of the organoids that changed unexpectedly during culturing. In some studies, brain organoids derived from different brain regions were merged to rebuild the brain, and the MIS may be used to monitor this process (Bagley et al., 2017). Overall, the MIS can help maintain cell viability during 2D and 3D culturing as well as be used to monitor the detailed processes during cell growth.

### 3.5 Label-free cell proliferation and viability analyses

**FIGURE 5:**
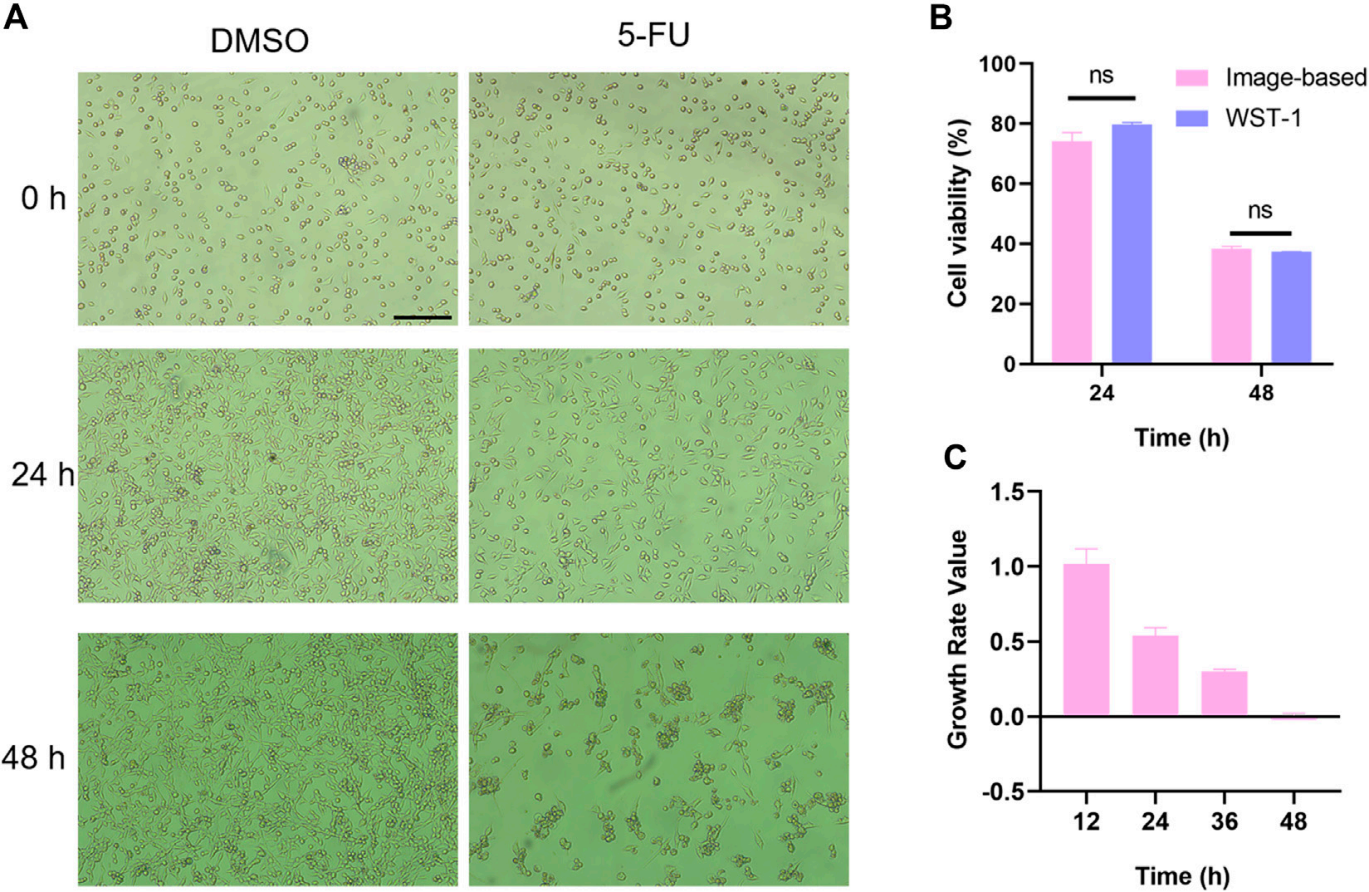
Label-free analysis of drug cytotoxicity on cells cultured in the MIS. **(A)** Representative bright-field images of DMSO-treated and 5-FU-exposed (1 μM) MC38 cells cultured in the MIS at different times post treatment. **(B)** Cytotoxic effects of 5-FU analyzed by an image-based method in the MIS and a colorimetric method in the Thermo-371 incubator at 24 h and 48 h post treatment. **(C)** Growth curve of the 1 μM of 5-FU-treated MC38 cells cultured in the MIS calculated based on cell confluence. Scale bar: 200 μm. ns: *p* > 0.05. DMSO: dimethyl sulfoxide; 5-FU: 5-fluorouracil; WST-1: water-soluble tetrazolium salt-1.

Accurate and rapid assessments of the chemotherapeutic effects on patient-derived cells are important in precision medicine. In general, end-point methods, such as the methyl thiazolyl tetrazolium (MTT), cell counting kit-8 (CCK-8), or proliferation marker staining (such as BrdU or Ki-67), are widely used for testing the cell viability (Gordon et al., 2018). However, these methods cannot track specific cell growths in real time and cannot distinguish between the cytostatic and cytotoxic drug effects (Mertens et al., 2023); here, we explored if the proposed MIS could be used to assess the drug sensitivity of the tumor cells by live cell imaging. Cells cultured in the MIS were imaged, and the cell viability was determined based on cell confluence (Figure 5A). These label-free cell proliferation and viability analyses allowed non-invasive monitoring of the cells in real time (Elson et al., 2023). Then, the therapeutic effects of chemotherapeutic agents, such as 5-fluorouracil (5-FU), irinotecan (CPT11), and oxaliplatin, were analyzed by this label-free method using the MIS. Similar effects of 1 μM of 5-FU were observed upon analyses by the label-free method and the WST-1 assay (Figure 5B). According to Hafner et al. (2016) and Elson et al. (2023), if the calculated GR is between 0 and 1, then the drug is considered to be cytostatic; if the GR is between −1 and 0, the drug is considered to be cytotoxic. Thus, the inhibitory effects of the drugs on cell growth, which gradually increased over time, were shown based on the label-free method (Figure 5C). Similarly, significant inhibitory abilities of CPT11 and oxaliplatin were shown based on real-time imaging (Supplementary Figure S5). In summary, using the MIS with a microscope enables label-free analysis of the therapeutic effects of chemotherapy drugs, thus displaying its potential in personalized therapy.

### 3.6 MIS provides a platform for monitoring cell migration

**FIGURE 6:**
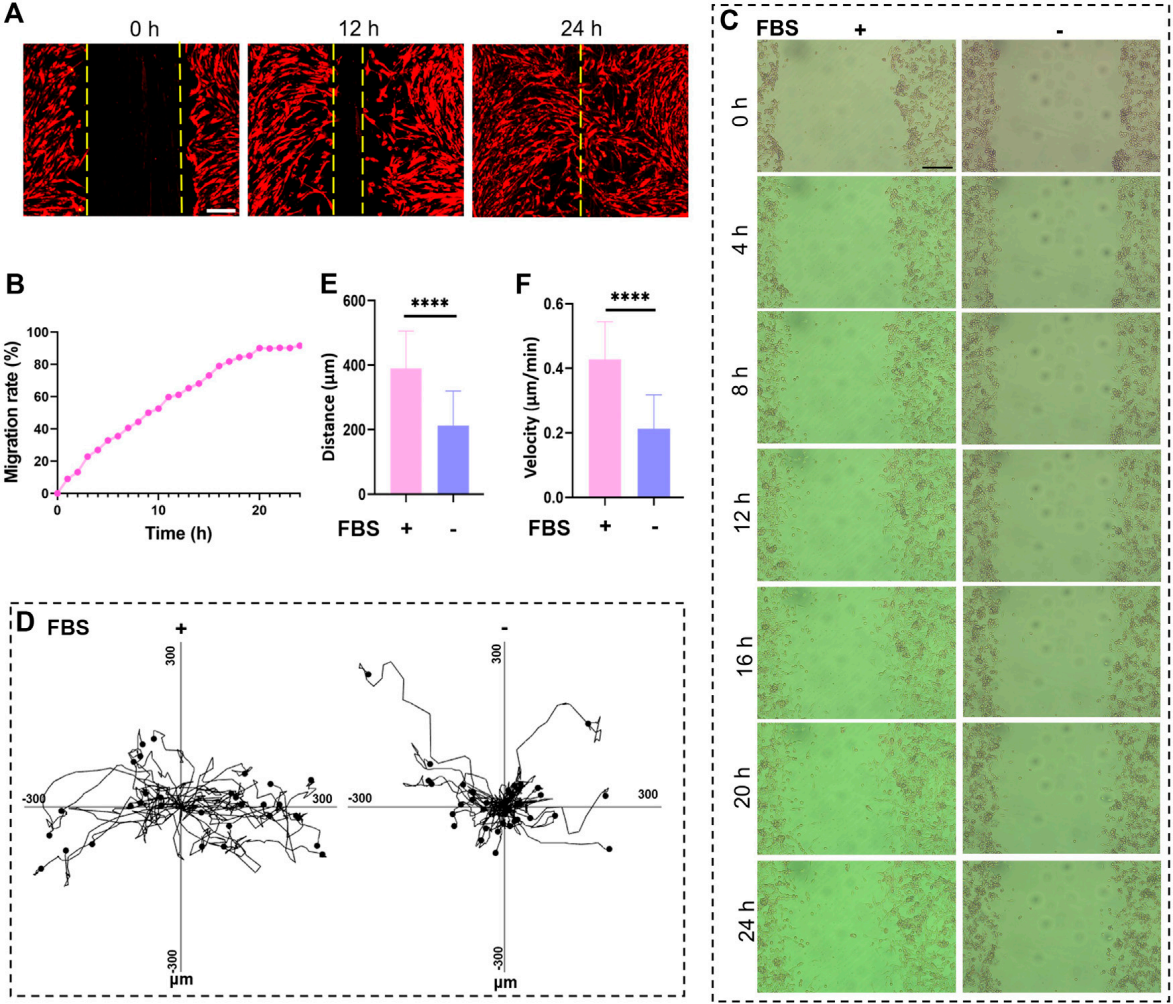
The microscope incubation system (MIS) was used to record cell migration. **(A)** Cancer-associated fibroblast (CAF) migrations in the MIS recorded at 0 h, 12 h, and 24 h. **(B)** Real-time migration of CAFs recorded every 1 h over 24 h. Bright-field images of cell migrations recorded at **(C)** various time points as well as the **(D)** movement trajectories, **(E)** movement speeds, and **(F)** movement distances of MC38 cells cultured in the MIS. Scale bar: 200 μm. DIL: 1,1′-dioctadecyl-3,3,3′,3′-tetramethylindocarbocyanine perchlorate; FBS: fetal bovine serum.

Since many cancer-related deaths are caused by tumor metastases, we explored whether the MIS could be used to evaluate cell migration and uncover the mechanisms of tumor metastases (Liang et al., 2007). Herein, we performed the scratch assay in the MIS, and cell migration was recorded by the MIS in real time. The cell migration rate was calculated based on the scratch area (Figures 6A,B). Moreover, tracking the migration of homogenous cell populations via live cell imaging allows following the paths of individual cells at the edges of a wound (Liang et al., 2007). MC38 cells with low mobility were chosen for monitoring single-cell migration; FBS was used to trigger cell migration from the scratches (Figure 6C). The trajectories of the migrating cells were clearly obtained with the MIS and imaging system (Figure 6D), and the migrating cells were observed to move faster and farther in the presence of FBS (Figures 6E,F). These findings show that the MIS can be used to monitor cell migrations, particularly those of individual cells, which are crucial for studying the leading edges of migrating cells and for testing drug effects on cell migration.

### 3.7 Real-time recording of immune-cell-mediated tumor cell death in the MIS

**FIGURE 7:**
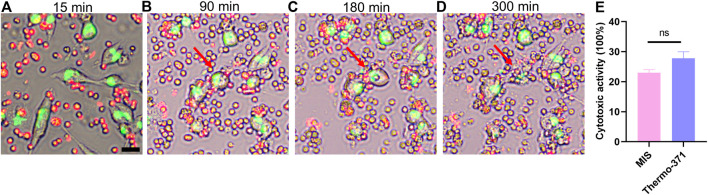
Co-culture of T cells and tumor cells in the MIS displayed the process of T-cell mediated tumor cells killing in real time. Typical images of T-cell mediated tumor cells killing, which indicated the process of physical contacting between T cells and tumor cells **(A)**, cytoplasmic granulation of tumor cells **(B)**, swelling **(C)** and lysis of tumor cells **(D)**. The T-cell was labeled by DIL (red) and the MC38 was labeled by DIO (green). **(E)** Cytotoxicity of T cells cultured in the MIS showed no significant differences compared with those cultured in the Thermo-371 basing on LDH analysis. Scan bar: 20 µm. ns: p > 0.05. DIL: 1,1′-dioctadecyl-3,3,3′,3′-tetramethylindocarbocyanine perchlorate, DIO: 3,3'-dioctadecyloxacarbocyanine perchlorate, LDH: lactate dehydrogenase.

Tumor immunotherapy is a kind of biotherapy that uses substances from living beings to help the immune system battle cancer (Kennedy and Salama, 2020). Understanding of immune cell killing (ICK) is crucial in this field, but numerous challenges were reported when modeling this process *in vitro*. Here, we assessed whether the MIS could be used to analyze the ICK process. As cytotoxic T lymphocytes (CTLs) killed the target tumor cells by secreting granzyme and perforin (Stinchcombe et al., 2001; Bálint et al., 2020), we cocultured CTLs isolated from the spleens and tumor cells in the MIS. Real-time imaging was performed every 15 min over 6 h, and the ICK process was recorded as shown in Figure 7A-D and Supplementary Video S2. The CTLs (green) actively moved and migrated toward the tumor cells (red); the CTLs attacked the tumor cells, leading to cell lysis and cell death. We then measured the cytotoxicity of the CTLs in the MIS and compared it with that in the Themo-371 incubator. No significant difference was found (*p* > 0.05, Figure 7E). Therefore, the MIS can be used to maintain the viability of suspended cells and record the ICK process in real time, which shows promise for advancing cancer immunotherapy.

## 4 Discussion

Long-term imaging of living cells and tissues is instrumental in revealing the cellular dynamics and functions (Baričević et al., 2023). Cell cultures with good statuses are fundamental for cell biology studies (Kreutzer et al., 2017), so stable environmental conditions in the incubator, such as temperature, pH, and humidity, are required during long-term live cell imaging (Ragazzini et al., 2019). However, standard incubators are often incompatible with many screening tools, which limits the experimental possibilities. There has been a trend toward the development of smaller incubators that can be placed atop a microscope stage. These mini incubators allow experiments to be performed with real-time imaging, recording equipment, and treatment (Rajan et al., 2018). The stage-top incubators developed by Okolab, Tokai Hit, and ibidi have been used to record live cells in many studies, but their high prices limit their universal use.

This study was aimed at designing and developing a mini MIS that could be used for real-time and long-term live cell imaging. To maintain a constant temperature of 37°C, a PID algorithm was used to set up the control feedback section composed of a sensor, a controller, and a heater. Specific materials with high thermal conductivities were used to ensure heat transfer efficiency, and a material with low thermal conductivity was chosen to decrease the heat loss from the system. Multiple heating membranes were integrated to warm the entire incubator uniformly. An internal humidifier was used to maintain a high-humidity environment in the incubator through water evaporation facilitated by an adjacent heater. Forced convection was used to enable external gas premixing and maintain stable gas concentration in the gas distribution module. A highly transparent quartz glass material was used for both the top and bottom covers of the incubator to enable clear imaging. Based on this design, the system temperature was measured as 37°C ± 0.16°C, while the gas concentration was maintained at 5% ± 0.13% by volume; further, the humidity of the system was maintained at above 90%. The incubator was suitable for use with most standard inverted microscopes, and its interior space was ideal for most cell culture dishes or plates. These parameters of the mini incubator are also comparable to or improvements over commercial stage-top incubators (Table 1).

**TABLE 1:** Comparisons of the key parameters of commercial stage-top incubators and the newly designed microscope incubation system (MIS).

	Okolab	Tokai Hit	ibidi	MIS
Dimensions (in mm)	160 × 110 × 29	187 × 146 × 27	127.5 × 85.5 × 25	160 × 130 × 29
Temperature accuracy	±0.1°C/± 0.3°C	±0.3°C	±0.2°C	±0.19°C
Gas accuracy	±0.1% vol	±0.1% vol	±0.5% vol	±0.13% vol

To determine whether the incubator could be used for cell culturing, the viability of the cells cultured in the MIS or a conventional incubator was compared via the WST-1 assay, LDH releasing assay, and calcein-AM/PI staining, which revealed no significant differences (*p* > 0.05). Organoid growth also displayed no significant differences (*p* > 0.05) when cultured in the mini or conventional incubators. Thus, it was considered that the MIS was ideal as a safe incubator for cell culture. When the MIS was used for long-term imaging of organoids, collapse and fusion was observed since day 6; this process occurred over less than 24 h, which often goes unnoticed without real-time imaging. The inflation and collapse of the organoids may be regulated by the mechanical forces in and self-organization abilities of the intestinal stem cells. Moreover, organoid fusion could be used to study the interactions between different brain regions (Bagley et al., 2017) and vascularized organoids (Sun et al., 2022). Thus, the mini incubator could be used to study the dynamic physiological processes during organoid culture.

During long-term culture and imaging, end-point methods are often not available for testing the cell viability. In this study, we tried to calculate the cell viability by cell confluence based on time-lapse images (Elson et al., 2023). The label-free method of measuring cell viability is easy to perform and does not interfere with continuous cell growth. Thus, the label-free method was applied to analyze the antidrug abilities of tumor cells, demonstrating the remarkable adaptability and versatility of the MIS. As this approach involves time-lapse imaging and sophisticated image analysis algorithms, the integration of an imaging system directly with the device should be studied in the future, which will enable the creation of an integrated platform for cell culture, imaging, and analysis.

It is known that many cancer deaths are often caused by tumor metastases, but it is unclear whether such metastases are the result of single-cell or collective cancer cell migrations. In this study, we not only recorded collective cancer cell migration in the wound healing assay but also traced single-cell movements using the MIS (Figure 6). Therefore, the mini incubator can potentially be used to study the mechanisms during cancer metastases. The CTLs play an essential role in target cell death through cellular interactions or paracrine effects (Weigelin et al., 2021). The process of target cell death often occurs over a short time, and it is difficult to record the entire cell killing process in a conventional incubator. The MIS offers a valuable platform to study the proliferation, migration, and cell–cell interactions in the process of T-cell-mediated cell killing, which highlights its potential in the field of immunotherapy.

In the present study, a uniform incubator was designed for specific investigations. An incubator that exerts negative pressure has been reported earlier to study the function of negative pressure on wound healing (Yamashiro et al., 2023). Incubators constructed without ferromagnetic materials are ideal for investigating the impacts of weak magnetic fields and low-intensity electromagnetic radiation on cell growth, proliferation, and differentiation (Krasts et al., 2010). In addition, a multitemperature incubator has been designed and reported to culture tissue samples required at different times (Williams, 1923). The oxygen content can be finely tuned by modulating the ratio of air to nitrogen to mimic various tissue oxygenation levels (Matthiesen et al., 2021). For our newly developed MIS, the effects of a hypoxia environment can be investigated by attaching an N_2_ cylinder in place of the air component in the system. More specific applications can also be studied in the future.

In conclusion, this study presents a newly developed MIS that exhibits comparable cell viability and confluence levels compared to a standard incubator. Real-time imaging in the MIS further supports the effectiveness of the mini incubator for analyzing cell growth, cell movements, and cell–cell interactions, which can be observed through long-term image and video captures. The MIS can potentially be used in dynamic studies in various fields, such as cell culturing, drug screening, cell therapy, and other cell-based assays. These research efforts could also enhance the development of more cost-effective instruments with space utilization and portability benefits for *in vitro* cell cultures, which have important implications in biomedical research and applications.

## Data Availability

The original contributions presented in the study are included in the article/Supplementary Material, and any further inquiries may be directed to the corresponding authors.
