# Supplementary material for: Establishment of the microscope incubation system and its application in evaluating tumor treatment effects through real-time live cellular imaging
Source: Front Bioeng Biotechnol. 2024 Aug 16;12:1447265. doi: 10.3389/fbioe.2024.1447265 (PMC11362064; doi:10.3389/fbioe.2024.1447265)
Supplement: Supplementary file 1 [file DataSheet1.docx]

Supplementary Material

**Establishment of** **microscope incubation system and its application in evaluation of tumor treatment effects by real time live cells imaging**

**Haiyang Yan, Tong Wu, Xinlu Li, Zhengyang Feng, Mingfeng Ge^*^, Lixing Zhang^*^ and Wen-fei Dong^*^**

*** Correspondence:** Mingfeng Ge: [gemf@sibet.ac.cn](mailto:gemf@sibet.ac.cn), Lixing Zhang: zhanglx@sibet.ac.cn, Wen-Fei Dong: [wenfeidong@sibet.ac.cn](mailto:wenfeidong@sibet.ac.cn).


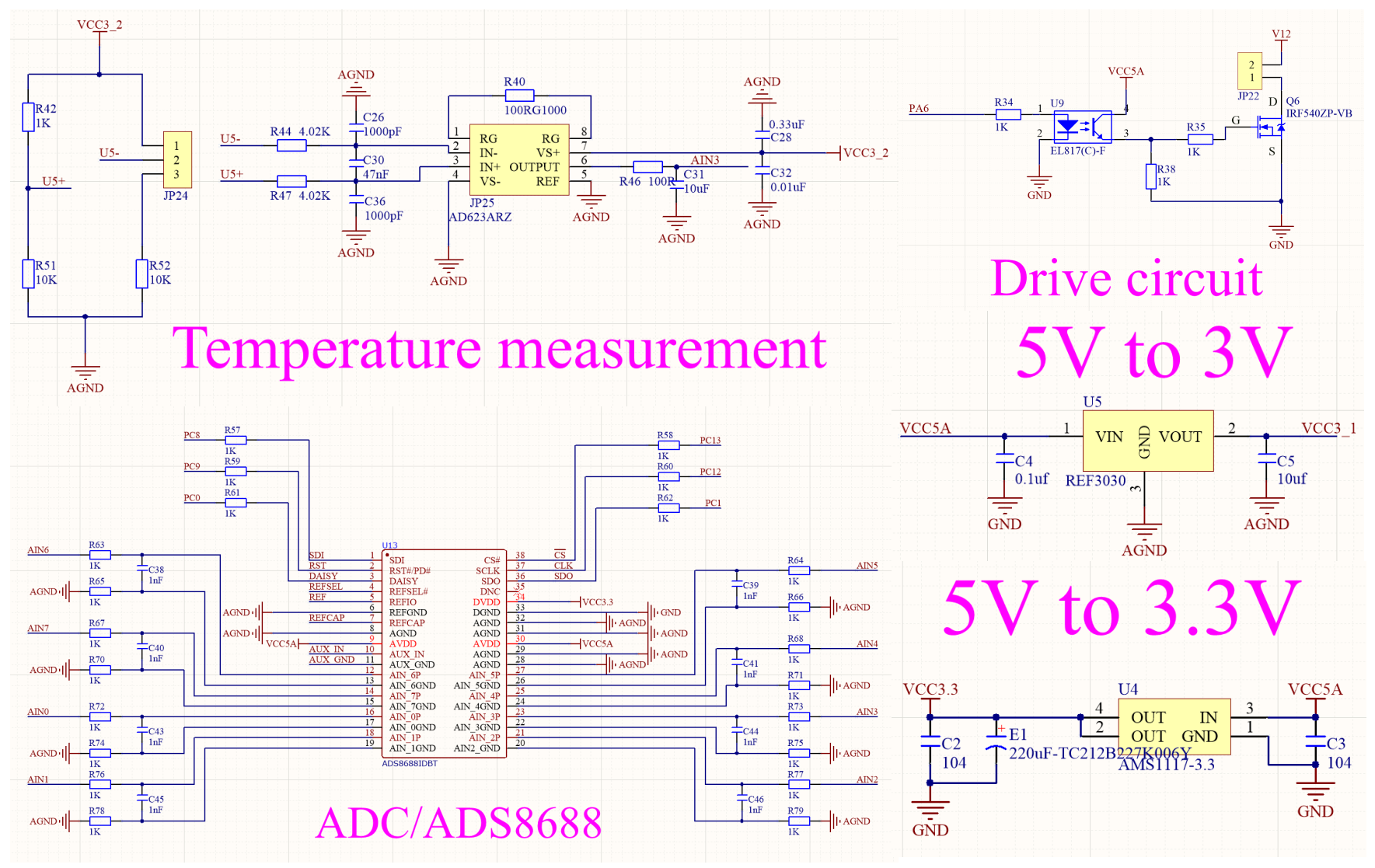


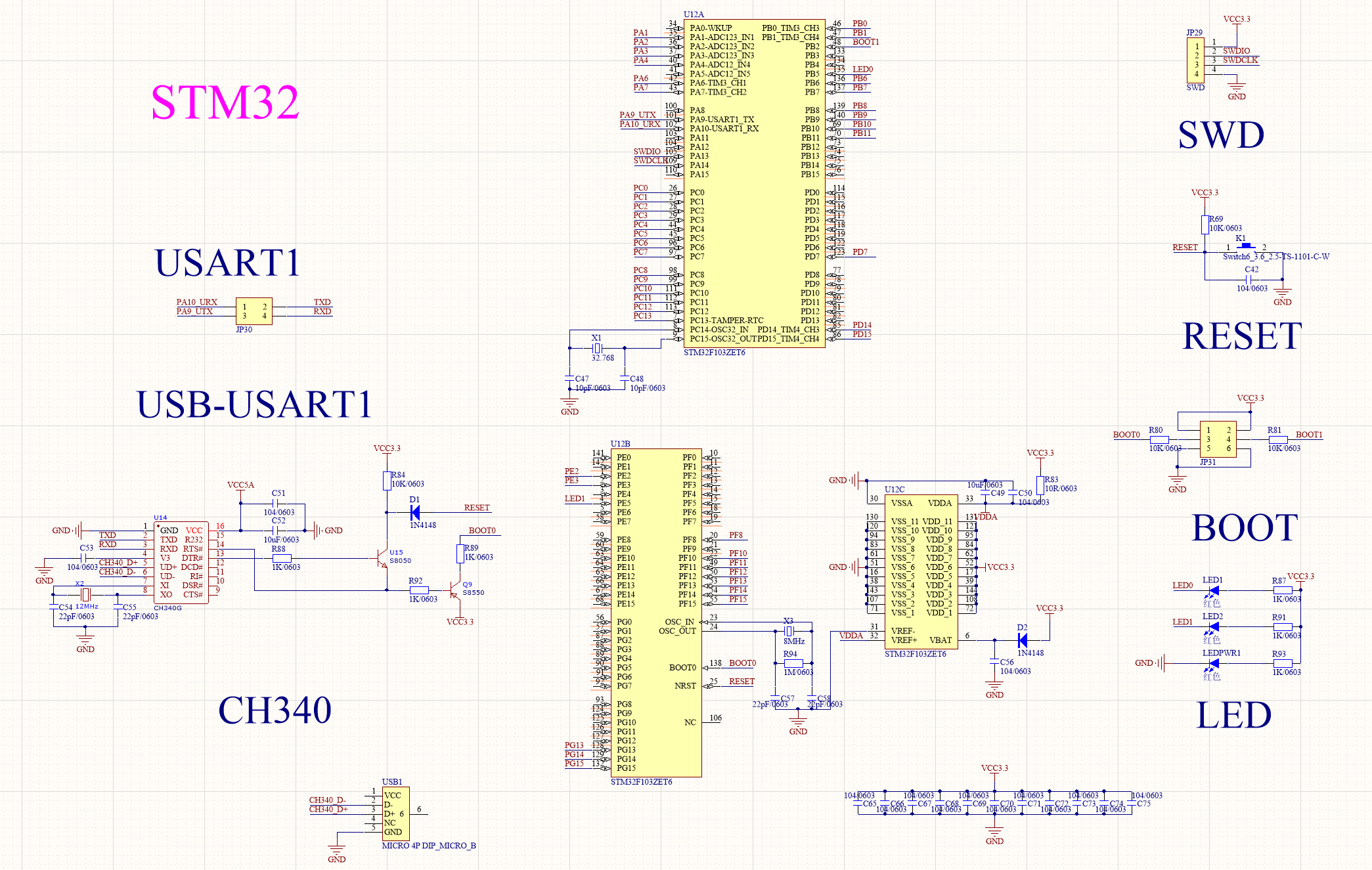


**Supplementary Figure 1.** The overall circuit schematic diagram of the control circuit board.


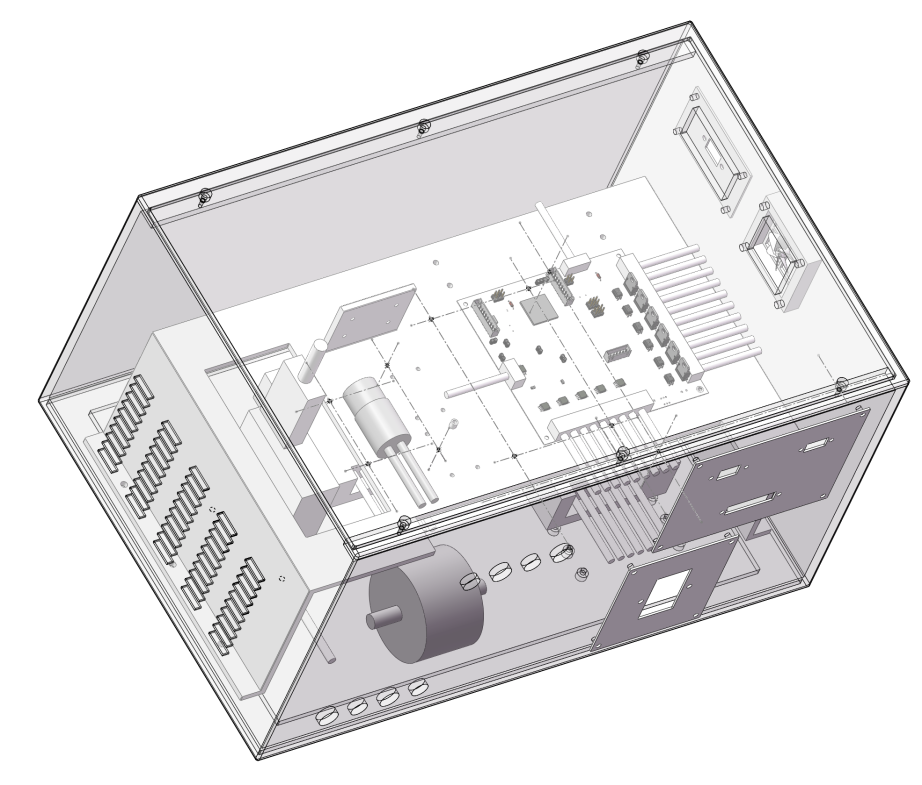


**Supplementary Figure 2.** The mechanical model of the component arrangement inside the control box.


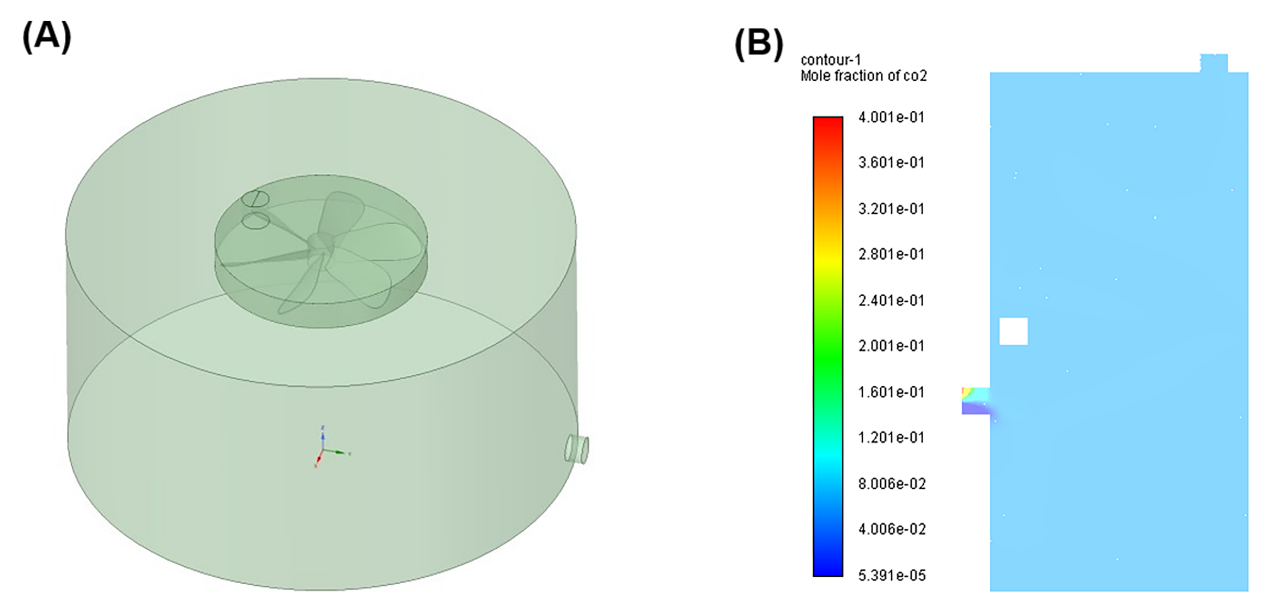


**Supplementary Figure 3.** Characterization of gas of the microscope incubation system (MIS). **(A)** Numerically computed fluid domain model in Space claim with the center of the bottom of the mixing chamber as the origin. **(B)** Simulation results for CO_2_ mass fraction in the plane at X = 0, high uniformity of gas distribution at different heights in the mixing chamber.


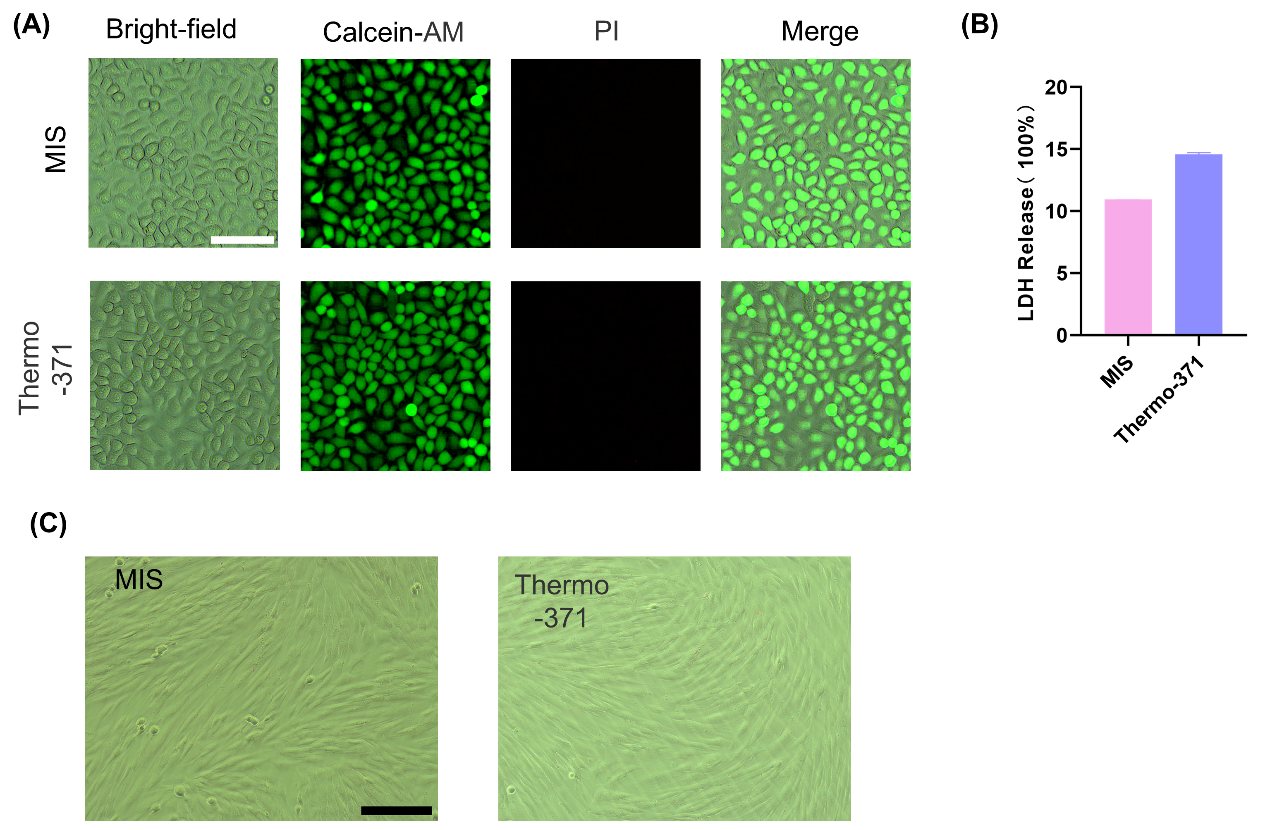


**Supplementary Figure 4.** Comparison of the growth status of cell in the microscope incubation system (MIS) and thermal 371 incubators. **(A)** Representative bright-field images of HeLa cells and fluorescent photographs of live and dead cells labeled by Calcein-AM/PI, the live cells were stained by Calcein-AM (green) and the dead cells were stained by PI (red). **(B)** The LDH release of HeLa cell cultured in (MIS) and thermal 371 incubators compared to the positive control. **(C)** Representative bright-field photographs of CAFs cultured in MIS or Thermo-371, both of which are morphologically similar, showing a shuttle-shaped. White scan bar: 100 μm; Black scan bar: 200 μm. LDH: lactate dehydrogenase; Calcein-AM: calcein acetoxymethyl ester; PI: propidium iodide; CAFs: Cancer associated fibroblasts.


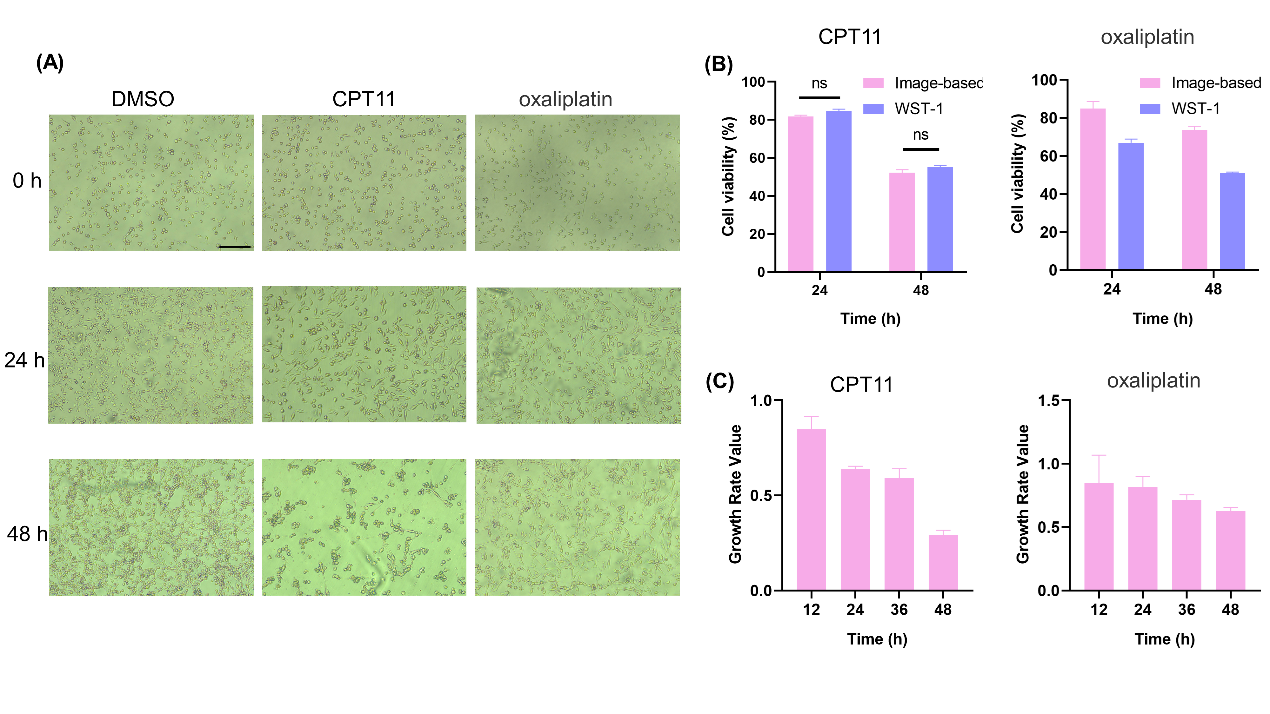
**Supplementary Figure 5.** The effect of CPT11 and oxaliplatin on MC38 cell analyzed by the MIS. **(A)** Representative brightfield images of DMSO-treated and CPT11 or oxaliplatin-exposed (100 μM) MC38 cell line at different time, the left panel was cell treated by DMSO, the middle panel was cell treated with CPT11 and the right panel was cell treated with oxaliplatin. **(B)** Relative cell viability values were measured by imaging-based and colorimetric methods at 24h and 48h, the left panel was cell treated with CPT11 and the right panel was cell treated with oxaliplatin. **(C)** Effect of CPT11 or oxaliplatin (100 μM) on the growth rate of MC38 cells at 12, 24, 36 and 48 hours, the left panel was cell treated with CPT11 and the right panel was cell treated with oxaliplatin. Scan bar: 200 μm. ns: P > 0.05. DMSO: dimethyl sulfoxide; CPT11: Irinotecan; WST-1: water-soluble tetrazolium salt-1.


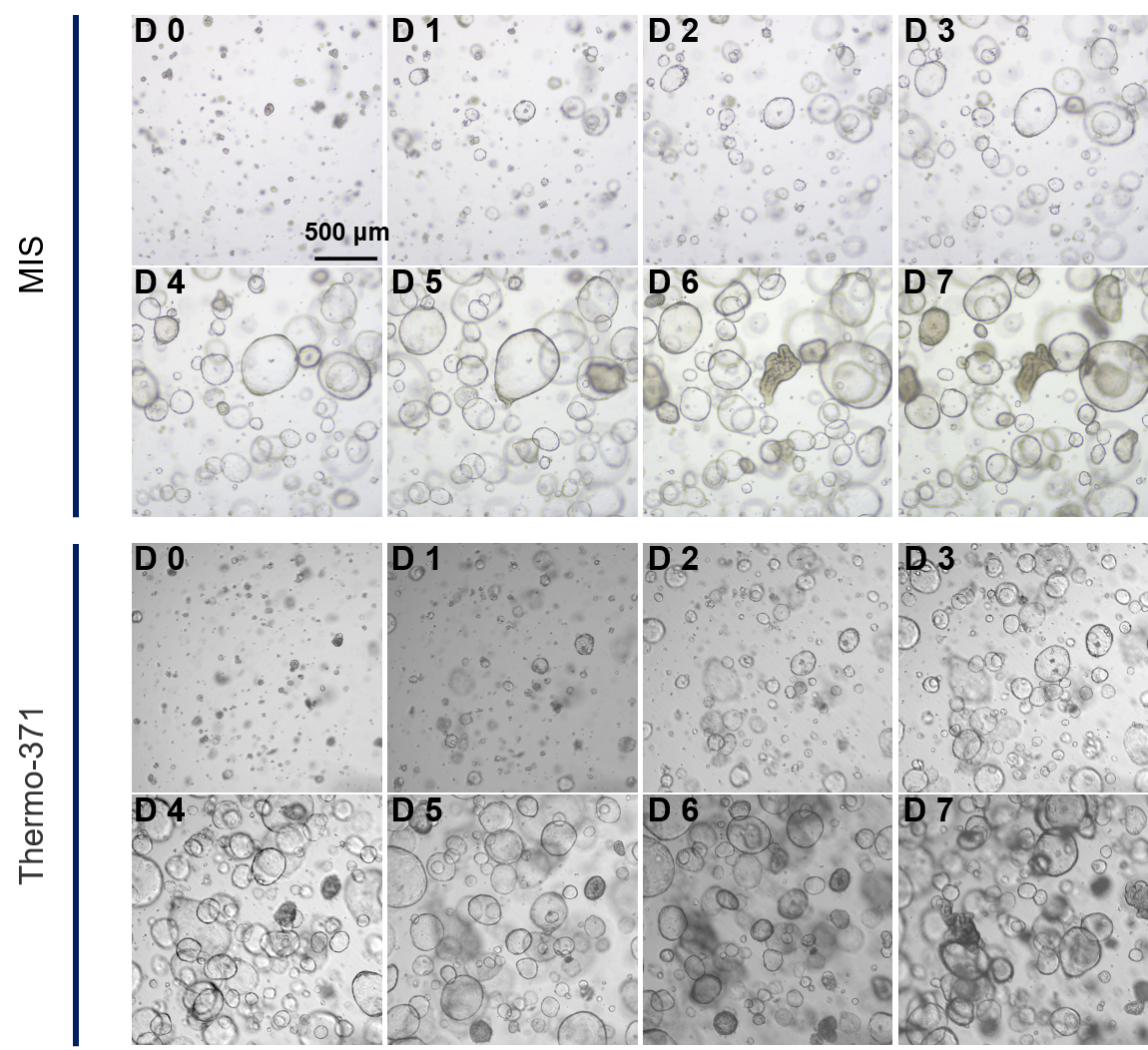


**Supplementary Figure 6.** Comparison of organoids cultured in MIS or Thermo-371 for different days. The upper panel showed organoids cultured in MIS, and the lower panel showed the organoids cultured in Thermo-371. No significant morphological differences were observed in organoid.

**Supplementary Video 1.** Growth video of organoids cultured in the microscope incubation system (MIS), which was made with images taken by the microscopy every 15 min from day 0 to day 7. Scan bar: 500 μm.

**Supplementary Video 2.** T cell and tumor cell were co-cultured in the microscope incubation system (MIS) and the cytotoxic effects of T cells was recorded by the microscopy every 15 min. Scan bar: 200 μm.
